# Influence of prenatal and early-life exposures on food allergy and eczema in infancy: a birth cohort study

**DOI:** 10.1186/s12887-019-1623-3

**Published:** 2019-07-17

**Authors:** Xiao Gao, Yan Yan, Guangyu Zeng, Tingting Sha, Shiping Liu, Qiong He, Cheng Chen, Ling Li, Shiting Xiang, Hongyan Li, Shan Tan, Qiang Yan

**Affiliations:** 1 Department of Epidemiology and Health Statistics, Xiangya School of Public Health, Central South University, Changsha, 410078 Hunan Province China; 2 Department of Maternal and Child Health, Kaifu District Health Bureau, Changsha, China

**Keywords:** Food allergy, Eczema, Prenatal and early-life exposures, Infants

## Abstract

**Background:**

Few prospective birth cohort studies are available on the effects of prenatal and early-life exposures on food allergy and eczema among Chinese children. The aim of this study was to evaluate the influence of prenatal and early-life exposures on food allergy and eczema during the first year of life in a prospective birth cohort study.

**Methods:**

This study was based on a prospective, observational birth cohort of 976 mother-child pairs in three Streets in Changsha, China from January to December 2015. Data on prenatal, early-life exposures and allergic outcomes were obtained from questionnaires collected at birth, and 1, 3, 6, 8, and 12 months of age. Multivariate logistic regression models were performed to estimate the effects of prenatal and early-life exposures on food allergy and eczema.

**Results:**

Common risk factors for food allergy and eczema in infancy were parental history of allergy, while moderate eggs consumption (3–4 times/week) during pregnancy was protective for both of them compared with low consumption (≤ 2 times/week). Factors only associated with food allergy were maternal aquatic products consumption during pregnancy, number of older siblings and age of solid food introduction, whereas factors only associated with eczema were maternal milk or milk products consumption during pregnancy, maternal antibiotic exposure during pregnancy, season of birth and antibiotic exposure through medication during the first year of life.

**Conclusion:**

Our study suggests that factors associated with food allergy and eczema are multifaceted, which involving hereditary, environmental and nutritional exposures. Furthermore, differential factors influence the development of food allergy and eczema in infants.

## Background

Food allergy is manifested in an unusual immunological reaction to a specific food or foods [1]. Eczema is a chronic, recurrent and inflammatory skin disease, which is often accompanied by itching [2]. The prevalence of both food allergy and eczema among children have risen evidently in the past decades [3, 4]. Although food allergy and eczema may not be fatal, these diagnoses are associated with an adverse impact on the quality of life [5, 6]. In addition, both of them are known as the earliest expressions of allergic diseases in infancy, and they are associated with increased risks of developing other allergies in later life, such as allergic rhinitis and asthma [7, 8]. Therefore, identifying the risk and protective factors for food allergy and eczema may help to develop specific and early preventative measures, and to reduce the prevalence of food allergy and eczema, even that of allergic diseases.

Food allergy and eczema often begin to appear very early in life, which implies that genetic predisposition, as well as prenatal and early childhood exposures might influence the development of these allergic outcomes. The roles of many prenatal and early-life exposure factors in food allergy and/or eczema development have been studied in developed countries. However, the reported results from these studies were inconsistent. For instance, some studies showed a positive association between the early introduction of solid food and allergic outcomes [9, 10], whereas other studies reported a negative association or no associations [11–13]. Furthermore, despite active researches on the association between maternal diet during pregnancy and childhood allergic outcomes such as asthma, allergic rhinitis, and wheeze [14–16], few have focused on childhood food allergy and eczema [14, 17, 18], suggesting a field that should be further investigated.

On the whole, there are few studies from prospective birth cohort on the incidence of childhood food allergy and eczema in developing countries like China, not to mention those that study the relationship between exposure factors and these allergic outcomes. On the other hand, since the prevalence and influencing factors of food allergy and eczema may vary by geographic regions and ethnicity, whether the influencing factors previously identified in Western infants are associated with the allergy outcomes among Chinese infants needs to be further examined. The aim of this study was to evaluate the influence of prenatal and early-life exposures on food allergy and eczema during the first year of life in a prospective, community-based birth cohort study in Changsha, China.

## Methods

### Study design and population

This study was based on a prospective, observational, birth cohort designed to study various exposures that could influence health and development in early life. A complete description of the cohort has been reported elsewhere [19]. Ethics approval for this study was granted by the Independent Ethics Committee Institute of Clinical Pharmacology, Central South University, Changsha, China (Project number: CTXY- 130041-3-2). Briefly, women who delivered live-born babies in three Streets of Kaifu District, Changsha City, had no history of mental illnesses or brain diseases, agreed to participate and provided their written informed consents, were recruited between Jan 1, 2015, and Dec 31, 2015. Eventually, 976 eligible mother-child pairs constituted the final birth cohort.

### Data collection

After delivery, baseline data regarding demographic characteristics and potential influencing factors for allergic outcomes were collected. Follow-up investigations collecting data on early-life exposures, infant feeding, and allergic outcomes were collected at 1, 3, 6, 8, and 12 months of age respectively with questionnaires. The infantile principal caregiver (usually mothers) completed questionnaires with a well-trained investigator during home visits at birth and at 12 months of age. Telephone interviews were performed at other time points.

### Definitions

The status of food allergy and eczema in the infant was assessed by a well-trained investigator and the definitions of these were standardized at each follow-up home visit and interview.

Food allergy: Due to ethical considerations, safety concerns and resource limitations, oral food challenges (OFC) were not performed. Infantile food allergy was determined by an affirmative answer to the following question: “Has your child ever had a doctor-diagnosed food allergy or experienced an allergic reaction immediately to a specific kind of food upon consumption as follows: swollen lips or face, urticaria, wheezing, vomiting, diarrhea, constipation)?” If the caregiver answered yes, the type of food involved was also ascertained.

Eczema: Infantile eczema was determined by an affirmative answer to the following question: “Has your child ever had a doctor-diagnosed eczema or had an itchy rash (excluding contact dermatitis) treated with topical steroid?”

An infant was deemed to have that specific allergic outcome when the question was answered affirmatively at any stage of the follow-up within 12 months.

### Exposures

The prenatal and early-life variables used in this study include: maternal age (continuous), pre-pregnancy BMI (continuous), maternal education (> 12 years; yes, no), multiple pregnancy (twin/triplet pregnancy; yes, no), caesarean section (yes, no), maternal active or passive smoking during pregnancy (yes, no), exposure to antibiotics during pregnancy (yes, no), post-partum depression at 1 month after childbirth (yes, no), family income per capita (> 5000 yuan/month; yes, no), parental history of allergy (yes, no), maternal milk or milk products consumption during pregnancy (≤ 2 times a week, 3–4 times a week, ≥ 5 times a week), eggs consumption (≤ 2 times a week, 3–4 times a week, ≥ 5 times a week), beans or bean products consumption (< 1 time a week, 1–2 times a week, 3–4 times a week, ≥ 5 times a week), nuts consumption (< 1 time a week, 1–2 times a week, 3–4 times a week, ≥ 5 times a week), aquatic products consumption (< 1 time a week, 1–2 times a week, ≥ 3 times a week), infant’s sex (male, female), birth weight (< 2500 g; yes, no), preterm birth (< 37 weeks of gestation; yes, no), season of birth (autumn-winter; yes, no), number of older siblings (0, ≥1), domestic pets kept at birth (yes, no), exclusive breast-feeding (≥ 6 months; yes, no), solid food introduced (< 6 months; yes, no), and exposure to antibiotics through medication during the first year of life (yes, no).

Post-partum depression at 1 month after childbirth was evaluated using the Edinburgh Postnatal Depression Scale (EPDS), with a score of > 10 indicating women with probable post-partum depression [20]. Parental history of allergy was defined as either the mother or the father has a history of food allergy, eczema, asthma or allergic rhinitis. Maternal diet during pregnancy was collected using a food frequency questionnaire after delivery. Mothers filled out their average intake frequency during pregnancy for each food item, with frequencies defined as never, < 1 time a week, 1–2 times a week, 3–4 times a week and ≥ 5 times a week. Some categories were combined due to small sample size. Aquatic products were defined as fish, shrimp, crab, and shellfish. When evaluating the relationship between antibiotic exposure through medication and allergic outcomes during the first year of life, the chronology of antibiotic exposure and food allergy/eczema was taken into consideration, respectively. Infants were considered exposed if the exposure was reported in a former or the same follow-up questionnaire as the outcome, while those who were exposed after the onset of the outcome were considered as unexposed.

### Statistical analysis

Data are presented as means ± SDs for continuous variables and frequencies with percentages for categorical variables. For univariate analysis, Independent *t* tests were used for continuous variables, while χ^2^ or Fisher exact tests were used for categorical variables. We fitted a multivariate logistic regression model for food allergy and eczema, respectively; infant’s sex and parental history of allergy as well as other exposure variables that were associated with any one of the two outcomes with a *P* value less than 0.2 in the univariate analysis were included in both models. All statistical analyses were conducted using SPSS version 18.

## Results

### Subject characteristics

**Fig. 1:**
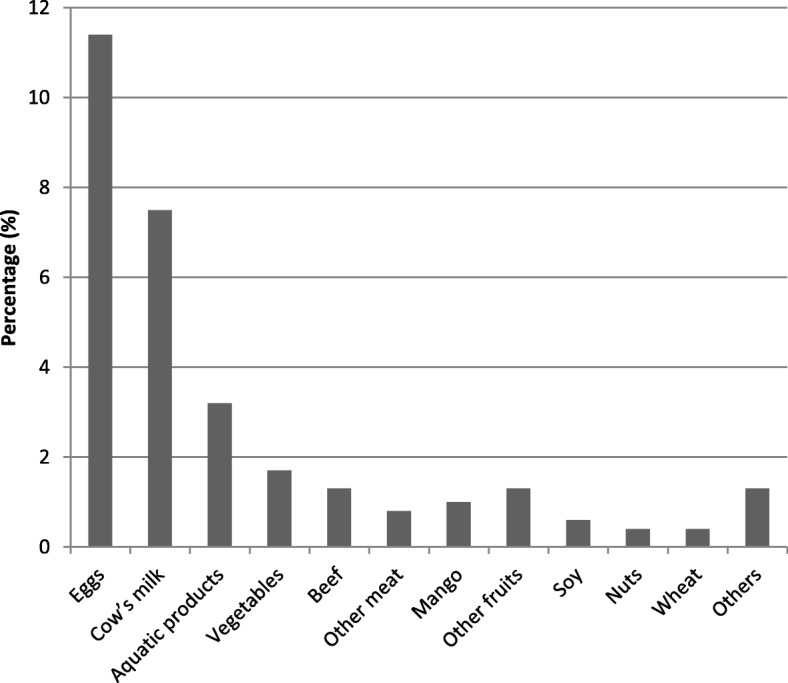
Percentage of infants classified as having an allergy to a specific kind of food during the first year of life

After a one-year follow-up, subjects with missing data on the outcome variables were excluded, leaving 903 infants (92.5%) included in the final analysis. Parental and infantile characteristics were detailed in Table 1. The mean age and pre-pregnancy BMI of mothers were 29.9 ± 3.8 years and 21.2 ± 3.0 kg/m^2^, respectively. In total, 259 (28.6%) and 188 (20.8%) women reported eating milk/milk products and eggs less than 3 times a week, 257 (28.5%) and 259 (28.7%) 3 to 4 times a week, 387 (42.9%) and 456 (50.5%) more than 5 times a week during pregnancy. 71.8% of mothers were primiparous and only 1.7% reported using antibiotics during pregnancy. A history of allergy was reported by 30.1% of parents. 41.3% of infants were born by caesarean section, and most of them were born at term, with normal birth weight. During the first year of life, 23.7% of infants were exposed to antibiotics through medication. 200 of 903 (22.1%) and 226 of 903 (25.0%) infants were reported to have food allergy and eczema during the first year of life. The top three causative foods were eggs, cow’s milk and aquatic products, with 103 (11.4%), 68 (7.5%) and 29 (3.2%) infants had eggs, cow’s milk, and aquatic products allergy, respectively (Fig. 1).

**Table 1:** Characteristics of the study subjects according to infants with and without food allergy or eczema in infancy

Variables	Total	Food allergy	No food allergy	*P* value	Eczema	No eczema	*P* value
No. of subjects	903	200	703		226	677	
Parental characteristics							
Maternal age (years) (*n* = 903)	29.9 ± 3.8	30.0 ± 3.8	29.9 ± 3.9	0.642^‡^	29.9 ± 3.6	29.9 ± 3.9	0.791^‡^
Pre-pregnancy BMI (kg/m^2^) (*n* = 883)	21.2 ± 3.0	21.3 ± 2.9	21.2 ± 3.1	0.687^‡^	21.2 ± 3.0	21.2 ± 3.0	0.957^‡^
Maternal education (> 12 years, %) (*n* = 903)	760 (84.2)	165 (82.5)	595 (84.6)	0.465^†^	189 (83.6)	571 (84.3)	0.799^†^
Multiple pregnancy (%) (*n* = 903)	23 (2.5)	5 (2.5)	18 (2.6)	0.962^†^	7 (3.1)	16 (2.4)	0.544^†^
Caesarean section (%) (*n* = 903)	373 (41.3)	91 (45.5)	282 (40.1)	0.172^†^	95 (42.0)	278 (41.1)	0.797^†^
Maternal active or passive smoking during pregnancy (%) (*n* = 903)	97 (10.7)	23 (11.5)	74 (10.5)	0.695^†^	28 (12.4)	69 (10.2)	0.356^†^
Exposure to antibiotics during pregnancy (%) (*n* = 903)	15 (1.7)	4 (2.0)	11 (1.6)	0.753^§^	8 (3.5)	7 (1.0)	0.017^§^
Domestic pets kept at delivery (%) (*n* = 903)	48 (5.3)	12 (6.0)	36 (5.1)	0.625^†^	13 (5.8)	35 (5.2)	0.735^†^
Post-partum depression at 1 month after childbirth (%) (*n* = 892)	52 (5.8)	16 (8.1)	36 (5.2)	0.125^†^	14 (6.2)	38 (5.7)	0.771^†^
Family income per capita (> 5000 yuan / month, %) (*n* = 902)	389 (43.1)	91 (45.5)	298 (42.5)	0.442^†^	92 (40.7)	297 (43.9)	0.396^†^
Parental history of allergy (%) (*n* = 903)	272 (30.1)	91 (45.5)	181 (25.7)	< 0.001^†^	94 (41.6)	178(26.3)	< 0.001^†^
Infantile characteristics							
Sex (male, %) (*n* = 903)	467 (51.7)	106 (53.0)	361 (51.4)	0.681^†^	115 (50.9)	352 (52.0)	0.773^†^
Birth weight (< 2500 g, %) (*n* = 900)	26 (2.9)	4 (2.0)	22 (3.1)	0.402^†^	5 (2.2)	21 (3.1)	0.483^†^
Preterm birth (< 37 weeks of gestation, %) (*n* = 898)	43 (4.8)	8 (4.0)	35 (5.0)	0.565^†^	11 (4.9)	32 (4.8)	0.949^†^
Season of birth (autumn-winter, %) (*n* = 903)	428 (47.4)	103 (51.5)	325 (46.2)	0.188^†^	115 (50.9)	313 (46.2)	0.225^†^
No. of older siblings (≥ 1, %) (*n* = 903)	255(28.2)	45 (22.5)	210 (29.9)	0.041^†^	54 (23.9)	201 (29.7)	0.094^†^
Exclusive breast feeding (≥ 6 months, %) (*n* = 903)	125 (13.8)	20 (10.0)	105 (14.9)	0.075^†^	26 (11.5)	99 (14.6)	0.240^†^
Solid food introduced (< 6 months, %) (*n* = 903)	720 (79.7)	171 (85.5)	549 (78.1)	0.022^†^	185 (81.9)	535 (79.0)	0.359^†^
Exposure to antibiotics through medication during the first year of life (%) (*n* = 903)^¶^	201 (22.3)^a^ 184 (20.4)^b^	43 (21.5)	158 (22.5)	0.770^†^	26 (11.5)	158 (23.3)	< 0.001^†^
Maternal diet during pregnancy							
Milk or milk products consumption (%) (*n* = 903)				0.335^†^			0.047^†^
≤ 2 times a week	259 (28.6)	56 (28.0)	203 (28.9)		51 (22.6)	208 (30.8)	
3–4 times a week	257 (28.5)	65 (32.5)	192 (27.3)		74 (32.7)	183 (27.0)	
≥ 5 times a week	387 (42.9)	79 (39.5)	308 (43.8)		101 (44.7)	286 (42.2)	
Eggs consumption (%) (*n* = 903)				0.341^†^			0.022^†^
≤ 2 times a week	188 (20.8)	47 (23.5)	141 (20.1)		55 (24.3)	133 (19.6)	
3–4 times a week	259 (28.7)	50 (25.0)	209 (29.7)		49 (21.7)	210 (31.0)	
≥ 5 times a week	456 (50.5)	103 (51.5)	353 (50.2)		122 (54.0)	334 (49.3)	
Beans or bean products consumption (%) (*n* = 903)				0.991^†^			0.716^†^
< 1 time a week	249 (27.6)	55 (27.5)	194 (27.6)		60 (26.5)	189 (27.9)	
1–2 times a week	302 (33.4)	67 (33.5)	235 (33.4)		72 (31.9)	230 (34.0)	
3–4 times a week	231 (25.6)	50 (25.0)	181 (25.7)		59 (26.1)	172 (25.4)	
≥ 5 times a week	121 (13.4)	28 (14.0)	93 (13.2)		35 (15.5)	86 (12.7)	
Nuts consumption (%) (*n* = 903)				0.497^†^			0.383^†^
< 1 time a week	217 (24.0)	50 (25.0)	167 (23.8)		55 (24.3)	162 (23.9)	
1–2 times a week	279 (30.9)	54 (27.0)	225 (32.0)		61 (27.0)	218 (32.2)	
3–4 times a week	191 (21.2)	42 (21.0)	149 (21.2)		48 (21.2)	143 (21.2)	
≥ 5 times a week	216 (23.9)	54 (27.0)	162 (23.0)		62 (27.4)	154 (22.7)	
Aquatic products consumption (%) (*n* = 903)				0.127^†^			0.249^†^
< 1 time a week	259 (28.7)	47 (23.5)	212 (30.2)		55 (24.3)	204 (30.1)	
1–2 times a week	381 (42.2)	95 (47.5)	286 (40.7)		101 (44.7)	280 (41.4)	
≥ 3 times a week	263 (29.1)	58 (29.0)	205 (29.2)		70 (31.0)	193 (28.5)	

Date are frequencies (percentage) or means ± SDs. BMI, body mass index; *n* represents the number of the study population with complete data

^†^χ^2^ Test; ^‡^Independent *t* test; ^§^Fisher exact test

^¶^There are two frequencies (percentage) of exposure to antibiotics through medication during the first year of life is because of the correction for the timing of exposure and food allergy (a) and for the timing of exposure and eczema (b), respectively

### Factors associated with food allergy and eczema

Infant’s sex and other exposure variables associated with food allergy and/or eczema during the first year of life with a *P* value < 0.2 in the univariate analysis were included in the multivariate logistic regression models. These variables included maternal antibiotic exposure during pregnancy, maternal milk or milk products, eggs and aquatic products consumption during pregnancy, parental history of allergy, caesarean section, post-partum depression at 1 month after childbirth, season of birth, number of older siblings, breast-feeding, age of solid food introduction and antibiotic exposure through medication during the first year of life.

Crude and adjusted ORs (95% CI) and *P* values were presented for exposure variables significantly associated with food allergy and/or eczema in the multivariate analysis (Table 2). Prenatal and early-life exposures significantly associated with an increased risk of food allergy during the first year of life were parental history of allergy [aOR = 2.45 (95% CI 1.75–3.42)], gestational aquatic products consumption [1–2 times/ week vs. < 1 time/week; aOR = 1.73 (95% CI 1.12–2.67)] and solid food introduction within the first 6 months of life [aOR = 1.76 (95% CI 1.12–2.76)]; while moderate gestational eggs consumption [3–4 times/week vs. ≤ 2 times/week; aOR = 0.61 (95% CI 0.38–0.99)] and infants had older siblings [aOR = 0.65 (95% CI 0.44–0.95)] showed a decreased risk of food allergy. Exposure variables significantly associated with a higher risk of developing eczema during the first year of life were parental history of allergy [aOR = 2.14 (95% CI 1.54–2.97)], moderate milk or milk products consumption [3–4 times/week vs. ≤ 2 times/week; aOR = 1.81 (95% CI 1.17–2.80)], maternal antibiotic exposure during pregnancy [aOR = 3.59 (95% CI 1.19–10.85)], as well as a borderline significance for children born in autumn-winter season [aOR = 1.38 (95% CI 1.00–1.91)]; while the main protective factors for eczema were moderate gestational eggs consumption [3–4 times/week vs. ≤ 2 times/week; aOR = 0.51 (95% CI 0.32–0.81)] and antibiotic exposure through medication during the first year of life [aOR = 0.44 (95% CI 0.28–0.70)].

**Table 2:** Odd ratios (95% CIs) for infants with food allergy and eczema in infancy

Variables	Food allergy (*n* = 200)				Eczema (*n* = 226)			
	Univariate		Multivariate^†^		Univariate		Multivariate^†^	
	OR (95% CI)	P	OR (95% CI)	P	OR (95% CI)	P	OR (95% CI)	P
No. of older siblings (≥1)	0.68 (0.47, 0.98)	0.042	0.65 (0.44, 0.95)	0.026				
Solid food introduced < 6 months	1.65 (1.07, 2.55)	0.023	1.76 (1.12, 2.76)	0.014				
Parental history of allergy	2.41 (1.74, 3.33)	< 0.001	2.45 (1.75, 3.42)	< 0.001	2.00 (1.46, 2.74)	< 0.001	2.14 (1.54, 2.97)	< 0.001
Aquatic products consumption								
< 1 time a week	1.00 (Reference)		1.00 (Reference)					
1–2 times a week	1.50 (1.01, 2.22)	0.043	1.73 (1.12, 2.67)	0.014				
≥ 3 times a week	1.28 (0.83, 1.96)	0.266	1.49 (0.89, 2.51)	0.132				
Eggs consumption								
≤ 2 times a week	1.00 (Reference)		1.00 (Reference)		1.00 (Reference)		1.00 (Reference)	
3–4 times a week	0.72 (0.46, 1.13)	0.150	0.61 (0.38, 0.99)	0.047	0.56 (0.36, 0.88)	0.011	0.51 (0.32, 0.81)	0.005
≥ 5 times a week	0.88 (0.59, 1.30)	0.510	0.82 (0.51, 1.33)	0.429	0.88 (0.61–1.29)	0.518	0.74 (0.47, 1.17)	0.201
Milk or milk products consumption								
≤ 2 times a week					1.00 (Reference)		1.00 (Reference)	
3–4 times a week					1.65 (1.10–2.48)	0.016	1.81 (1.17, 2.80)	0.008
≥ 5 times a week					1.44 (0.98–2.11)	0.061	1.35 (0.86, 2.10)	0.194
Exposure to antibiotics during pregnancy					3.51 (1.26–9.80)	0.016	3.59 (1.19, 10.85)	0.024
Exposure to antibiotics through medication during the first year					0.43 (0.27–0.67)	< 0.001	0.44 (0.28, 0.70)	< 0.001
Season of birth (autumn-winter)					1.21 (0.89–1.63)	0.226	1.38 (1.00, 1.91)	0.052

Crude and adjusted ORs(95% CI) and P values were presented for exposure variables significantly associated with food allergy and/or eczema in the multivariate analysis

^†^Infant’s sex and other exposure variables associated with food allergy and/or eczema during the first year of life with a *P* value < 0.2 in the univariate analysis were included in the multivariate logistic regression models

## Discussion

In this prospective birth cohort, the cumulative incidence of food allergy during the first year of life was 22.1%, which is higher than the prevalence of 11.0% [21] in the HealthNuts cohort study in Melbourne, Australia. The following reasons may help to explain in part this discrepancy. The HealthsNuts study reported the point prevalence in infants at 12 months old based on challenge-confirmed food allergy to egg white, peanut, and sesame, whereas our study counted the cumulative incidence in infants from birth to the age of 12 months based on parent report of a doctor diagnosis or an allergic reaction to any food. However, our estimate of food allergy is significantly lower than the previously reported prevalence of 40.0% in a pediatric university hospital in South African, mainly because the latter study was based on a population aged 6 months to 10 years who present with atopic dermatitis [22]. The cumulative incidence of eczema during the first year of life was 25.0% in our cohort, which is close to the previously reported percentage in the HealthsNuts study (28.0%) [23] and to that of another birth cohort study in Japan (27.9%) [24]. But it is significantly higher than the cumulative incidence reported by Cheng et al. [25] in a birth cohort study in Singapore (12.7%), which is mainly because the definition of eczema they used was only based on physician diagnosis, while our study was based on parent report of a doctor diagnosis or itchy rash treated with topical steroid. Therefore, the studies differ considerably in outcome definitions, length of follow-up, study population, and study design, which could account for the inconsistent results. Generally, the status of food allergy and eczema in infants in our cohort is quite disturbing, which indicates that healthcare practitioners should pay more attention to these serious health problems.

Consistent with previous studies, our study revealed an important role of parental history of allergy in the development of infantile food allergy (FA) and eczema [13, 24, 26]. Peters et al. identified one eczema phenotype and three food allergy phenotypes in the HealthsNuts study involving 5276 12-month-old infants and noted that family history of allergy was a risk factor for all phenotypes [26]. But a study consisted of 440 FA patients (FA group) and 332 non-FA patients (non-FA group) in 0- to 2-year-old children in Aichi, Japan, did not find statistical significance in the difference between the two groups in parental history of allergy, probably because they recruited both the case group and the control group from an allergy clinic [27]. Another prospective birth cohort study in Japan discovered that children with eczema in the first of life were more likely to have a parental history of allergic diseases compared with children without eczema [24]. Hence, heredity is a strong predictor of childhood food allergy and eczema.

There are limited studies focusing on the influence of maternal diet during pregnancy on childhood food allergy and eczema [14, 17, 18]. A longitudinal birth cohort study in the UK suggested that high maternal fish intake (≥ 1/week vs. never) during pregnancy was associated with a reduced risk of doctor-confirmed eczema in the offspring at age 5 years [14]. Pelé et al. conducted a questionnaire-based cohort study in Brittany, France, and showed that maternal pre-parturition shellfish consumption at least once a month increased the risk of childhood food allergy before age 2 compared with consumption less than once a month. However, they also observed that maternal pre-parturition fish consumption was not significantly associated with food allergy and eczema in their offspring [17]. In this study, we found that gestational aquatic products consumption once or twice a week was associated with an increased risk of infantile food allergy compared with consumption less than once a week. Future studies are needed to subdivide these aquatic products and assess separately the specific effects of different categories of aquatic products intake, since differential mechanisms may exist. A German prospective birth cohort study by Sausenthaler et al. [18] noted that high maternal intake of eggs (> 33% of all subjects) during pregnancy had a protective effect for eczema, but statistical significance was not reached. They also discovered no association between maternal milk intake during pregnancy and the development of food sensitization and eczema in the offspring at the age of 2 years. To the best of our knowledge, no previous study has suggested that maternal moderate eggs consumption (3–4 times/week vs. ≤ 2 times/week) during pregnancy is protective for infantile food allergy and eczema, and moderate milk or milk products consumption (3–4 times/week vs. ≤ 2 times/week) is associated with an increased risk of infantile eczema. Further large-scale, prospective, mother-child cohort studies are needed to corroborate these findings and researches on the possible biologic mechanisms underlying these findings are also essential.

Studies examining the impact of early introduction of solid food on food allergy show less consistent results. Some studies suggested inverse associations [11] or no associations [12] between early introduction of solid food and food allergy, whereas another birth cohort study in the UK, reported that increased age of solid introduction was significantly associated with a reduced risk of food hypersensitivity [10]. In the present study, we found that infants with solid food introduction before 6 months had a 1.76 fold the risk of developing food allergy compared with an introduction from 6 months. The World Health Organization recommends that infants should be exclusively breastfed within 6 months, and followed by the introduction of solid food at 6 months [28]. Our results supported the above recommendation. It is considered that infant’s gastrointestinal tract has not been sufficiently developed before 6 months and early introduction of solid food is likely to cause food allergy symptoms, due to the high intestinal permeability and increased food antigens uptake. In line with the results from previous studies, our study found that the presence of older siblings was associated with a lower risk of developing food allergy [29, 30]. A possible explanation for the protective association of the presence of older siblings is because of increased exposure to microbial stimulation in early childhood resulting from close contact with siblings. In addition, several studies have proposed that exposure to certain infections may protect against allergies [31, 32].

Few studies have addressed the influence of maternal antibiotic exposure during pregnancy on eczema in their offspring [33, 34]. A birth cohort study involving 411 children born of mothers with a history of asthma in Denmark, did not find a significant association between prenatal antibiotic exposure (third trimester) and early childhood eczema [34]. However, similar to our finding, Dom et al. discovered that prenatal antibiotic exposure increased the risk of childhood eczema in a birth cohort in Belgium [33]. The biologic mechanisms behind this association are not yet clear. A study indicated that maternal antibiotic exposure during pregnancy might alter fetal skin and gut microbiota [35], while Dom et al. argued that stress may play an important role [33]. The role of early-life antibiotic exposure through medication in the development of eczema remains controversial. Some studies reported early-life antibiotic exposure increased odds of eczema [36, 37], whereas another study did not find any significant association between them [38]. However, in contrast to these studies, our study observed an inverse association between antibiotic exposure through medication during the first year of life and infantile eczema, which confirmed the association found by Dom et al. [33]. This inconsistency in observations is probably because maternal antibiotic exposure during pregnancy was simultaneously adjusted in the multivariate analysis and the chronology of antibiotic exposure and eczema was also taken into consideration in our studies. Our study also found that children born in autumn or winter seasons were associated with an increased risk of developing infantile eczema compared with those children born in spring or summer seasons, with a borderline significance. Kuzume et al. [39] found that the cumulative sunshine amount during the 3 months before and after birth was negatively associated with the incidence of eczema. Furthermore, Lockett et al. [40] reported a similar finding that autumn birth increased the risk of eczema compared with spring birth and indicated that season-associated DNA methylation could play a harmful role in the development of eczema.

To our knowledge, this is the first prospective, community-based birth cohort study to evaluate the influence of prenatal and early-life exposures on food allergy and eczema in infancy in China. Selection bias was low due to the good compliance to follow-up. Meanwhile, the present study also has several limitations. A notable one is that measures of food allergy and eczema were obtained by questionnaires, without clinical examination or without objective assessment of food allergy through skin prick test or oral food challenge. Although some large-scale birth cohort studies have used validated questionnaires to assess the presence of food allergy or eczema [17, 24, 25, 33], these methods might raise non-differential misclassification. However, infants are offered five routine state-mandated health checks in China at 1, 3, 6, 8, and 12 months of age respectively. Therefore, the presence of food allergy and eczema were mainly evaluated by parent report of a doctor diagnosis in our study. Questionnaires were used to collect the data, which may lead to recall bias. However, the prospective follow-up at very close intervals enabled us to minimize recall bias during data collection. Most parents are sensitive to the health of their babies, also helping in reducing recall bias. Secondly, the study population only comprised of infants from the urban districts of Changsha, so the conclusions might not be generalizable to all Chinese infants. Lastly, we only evaluated the influence of prenatal and early-life exposures on food allergy and eczema in infancy, and some allergic diseases such as allergic rhinitis and asthma were not included in this study. However, allergic rhinitis and asthma often develop later in childhood and are not common among infants, which is the focus of the present study.

## Conclusions

Our study suggests that factors associated with food allergy and eczema are multifaceted, which involving hereditary, environmental and nutritional exposures. Furthermore, differential factors influence the development of food allergy and eczema in infants. Identifying the risk and protective factors for food allergy and eczema may help to develop specific and early preventative measures and to reduce the prevalence of food allergy and eczema, even that of allergic diseases.

## Data Availability

The datasets analyzed specifically for use in this study are not publicly available due to on-going research, but reasonable requests for data can be made to the corresponding author at the end of the research.

## Abbreviations

aOR: Adjusted odds ratios
BMI: Body mass index
CI: Confidence intervals
EPDS: Edinburgh Postnatal Depression Scale
OFC: Oral food challenges
SD: Standard deviations
SPSS: Statistical package for social science
